# First-line pembrolizumab in patients with advanced non-small cell lung cancer and high PD-L1 expression: real-world data from a Spanish multicenter study

**DOI:** 10.3389/fonc.2024.1510278

**Published:** 2024-12-17

**Authors:** Aida Piedra, Sergio Martínez-Recio, Ainhoa Hernández, Teresa Morán, Edurne Arriola, Jordi Recuero-Borau, Manuel Cobo, Patricia Cordeiro, Joaquín Mosquera, Manuel Fernández, Rosario García-Campelo, Antonio Calles, Rosa Álvarez, María Zapata-García, Dolores Isla, Ana Callejo, Patricia Iranzo, Jorgina Serra-López, Andrés Barba, Ivana Sullivan, Enriqueta Felip, Margarita Majem

**Affiliations:** ^1^ Medical Oncology Department. Hospital de la Santa Creu i Sant Pau, Barcelona, Spain; ^2^ Department of Medicine, Universitat Autònoma de Barcelona (UAB), Barcelona, Spain; ^3^ Medical Oncology Department, ICO Badalona, Hospital Universitario Germans Trias i Pujol, Barcelona, Spain; ^4^ Medical Oncology Department, Hospital del Mar – Centro de Investigación Biomédica en Red - Cáncer (CIBERONC), Barcelona, Spain; ^5^ Medical Oncology Department, Hospital Regional Universitario Virgen de la Victoria (IBIMA), Málaga, Spain; ^6^ Medical Oncology Department, Complejo Hospitalario Universitario de A Coruña, A Coruña, Spain; ^7^ Medical Oncology Department, Hospital Universitario Gregorio Marañón, Madrid, Spain; ^8^ Medical Oncology Department, Hospital Universitario Lozano Blesa, Zaragoza, Spain; ^9^ Medical Oncology Department, Hospital Universitario Vall d´Hebron – VHIO, Barcelona, Spain

**Keywords:** pembrolizumab, non-small cell lung cancer, first-line, predictive factors, high PD-L1 expression, immune check-point inhibitors

## Abstract

**Introduction:**

Pembrolizumab stands as a first-line option for patients with advanced non-small cell lung cancer (NSCLC) and high programmed death-ligand 1 (PD-L1) expression (PD-L1 ≥50%). Several factors such as antibiotic exposure, low body mass index (BMI), certain metastatic location or poor performance status may influence outcomes.

**Methods:**

We conducted a multicenter retrospective analysis in a cohort of patients with advanced high PD-L1 expression NSCLC treated with first-line pembrolizumab in clinical practice. We sought to evaluate clinical outcomes according to several factors.

**Results:**

Among the 494 included patients, median age was 67.29 years, 77% were male, 54% and 38% were former or current smokers, respectively; 84% had an Eastern Cooperative Oncology Group (ECOG) Performance Status (PS) of 0-1, and 48% had a BMI of <25. 32% of patients had bone metastases, 32% brain metastases and 16% liver metastases. 35% of patients had exposure to antibiotics (AB), 44% to corticosteroids and 62% to proton pump inhibitors (PPi). With a median follow-up of 14.3 months, the median overall survival (OS) and progression-free survival (PFS) were 15.9m (95% CI 13.1 to 18.8) and 9.9m (95% CI 7.7 to 12.1), and the overall response rate (ORR) was 43%. After univariate analysis, median OS in patients with ECOG-PS 0 vs. 1 vs. 2 was 36.7m vs. 14.8m vs. 2.7m (p<0.001). Median OS in patients who received treatment with corticosteroids vs. patients without exposure was 11.4m vs. 22.3m (p<0.001). After multivariate analysis, corticosteroid exposure (HR 1.41) and ECOG-PS (HR 2.40) maintained a prognostic impact.

**Discussion:**

First-line pembrolizumab outcomes in advanced high PD-L1 expression NSCLC patients could be negatively influenced by corticosteroid exposure or poor ECOG-PS.

## Introduction

Pembrolizumab, a programmed death (PD)-1 inhibitor, stands as a first-line option for patients with advanced non-small cell lung cancer (NSCLC) patients and a high programmed death-ligand 1 (PD-L1) expression [tumor proportion score (TPS) ≥50%], showing superior overall survival (OS), progression-free survival (PFS) and overall response rate (ORR) compared to chemotherapy, with better toxicity profile (1, 2). Results after 5 years of follow-up have been reported, with a median OS of 26.3 months and 31.9% of patients alive at 5 years (3). In addition, several real-world studies have confirmed these results in clinical practice (4–7). The PD-1 inhibitor cemiplimab, and the PD-L1 inhibitor atezolizumab have also shown efficacy in first-line setting with high PD-L1 expression (8, 9).

Of note, randomized clinical trials with pembrolizumab and chemotherapy have also shown OS benefit regardless of PD-L1 status, including patients with PD-L1≥50% expression (10, 11) and, more recently, results from clinical trial EMPOWER-Lung 3 also confirm better outcomes with cemiplimab and chemotherapy in patients with PD-L1≥50% (12). Other combinations of chemotherapy and anti-cytotoxic T lymphocyte-associated protein 4 (CTLA-4) and anti PD-(L)1 have also shown similar outcomes but with higher rates of adverse events (13, 14).

However, patients with potential negative predictive factors such as poor Eastern Cooperative Oncology Group (ECOG) Performance Status (PS) (15), advanced age or receiving concomitant treatments with immune-modulating effects, such as antibiotics, corticosteroids or proton pump inhibitors (PPi) (16) are usually underrepresented in immunotherapy clinical trials. Recent research has also suggested that other factors such as a low body mass index (BMI) (17, 18) or certain metastatic sites (19) can also negatively impact on the efficacy of first-line pembrolizumab.

As there are several treatment options available for patients with advanced NSCLC and high PD-L1 expression, many efforts have been made to identify predictive factors that may help to select the best strategy in this scenario. Two meta-analyses have suggested that chemo-immunotherapy may improve OS and PFS compared to immunotherapy alone (20, 21) in some subgroups of patients, such as women or never-smokers.

The objective of our study was to evaluate the real-world outcomes of patients with advanced NSCLC and high PD-L1 expression receiving first-line pembrolizumab therapy and to assess potential predictive factors in this population.

## Materials and methods

This multicenter retrospective analysis included patients with advanced NSCLC with a PD-L1 ≥50% that had received at least one dose of first line pembrolizumab monotherapy outside of clinical trials between August 1^st^ 2017 and January 1^st^ 2023. Patients had to be treatment-naive or have a tumor relapse ≥6 months after curative treatment and have not received previous immunotherapy as part of their treatment. Sample size was not restricted due to the exploratory nature of the study. Data were anonymized at inclusion in the data base and collected from medical records. The data cut-off was June 30^st^ 2023 to ensure a minimum follow-up of six months. The study was approved by a local ethics committee and confirmed by other institutions.

PD-L1 expression was determined by immunohistochemical staining in histological or cytological samples from primary tumors, lymph nodes or distant metastases, in each institution. Samples were considered valid if ≥100 viable cells were analysed.

Patient data and concomitant treatments were recorded from the medical history. Exposure to antibiotics, corticosteroids and PPi was considered during treatment and within four weeks before starting immunotherapy.

ORR and PFS were assessed by investigators using Response Evaluation Criteria in Solid Tumors (RECIST) version 1.1 (22) and iRECIST (23). Best response was categorized as complete response, partial response, stable disease and progressive disease (22, 23). Patients treated beyond radiographic progression were also recorded. PFS was defined as the time from the first dose of pembrolizumab to progression or death, and patients without disease progression were censored at the time of the last disease assessment. OS was calculated from the first dose of pembrolizumab until death. Patients who were still alive at the time of data analysis were censored at the time of last contact.

Descriptive statistics were used to report baseline characteristics of the population. Kaplan-Meier was used to estimate survival, and the long-rank test was used to compare median survival. Multivariate analyses were performed using Cox regression assuming proportional hazards. Two-sided p-values and 95% confidence intervals (CI) were used, with a prespecified <0.05 as significant. IBM Statistic SPSS version 25 software was used for the analyses.

## Results

494 patients were included from eight institutions. Patient clinicopathological characteristics are summarized in **Table 1**. White men, <75 years, former smoker, with a BMI ≥25, ECOG-PS of 1 and non-squamous histology were predominant in our population.

**Table 1:** Clinical and pathological characteristics.

	N/Median	%/Range
Age (years)		
<75	392	79
≥75	102	21
Median age	67.3	26.4 - 89.4
Sex		
Male	379	77
Female	115	23
Race		
White	488	99
Asian	4	<1
Black	2	<1
Smoking habit		
Former smoker	268	54
Current smoker	188	38
Never smoker	38	8
BMI (kg/m2)		
<18,5	22	4
18,5-24,9	206	42
≥25	236	48
Unknown	30	6
ECOG-PS		
0	138	28
1	269	55
2	81	16
3	5	1
Unknown	1	<1
AB exposure		
No	319	65
Yes	175	35
Oral	110	22
Intravenous	63	13
Unknown	2	<1%
Corticosteroid exposure		
No	277	56
Yes	217	44
Oral	169	34
Intravenous	44	9
Unknown	4	1
Reason for treatment with corticosteroids		
irAEs	57	12
Management of comorbidities/symptom	153	31
Unknown	7	1
PPi exposure		
No	186	38
Yes	308	62
Bone metastases		
No	336	68
Yes	158	32
CNS metastases		
No	388	78
Yes	105	21
Unknown	1	<1
Liver metastases		
No	417	84
Yes	77	16
Histology		
Non-squamous	373	76
Squamous	114	23
NOS	7	1
PD-L1 (%)		
≥50-≤59	182	37
>60-<90	187	38
≥90	125	25

BMI, body mass index; ECOG-PS, Eastern Cooperative Oncology Group-Performance Status; AB, antibiotic; irAEs, immune-related adverse events; PPI, proton pump inhibitor; CNS, central nervous system; NOS, No otherwise specified; PD-L1, programmed death-ligand 1.

PD-L1 expression was determined using 22C3 antibody (N=258, 52%), SP 263 antibody (N=164, 33%), 28-8 antibody (N=65, 13%) or Ventana SP142 antibody (N=1, <1%). Samples were obtained from primary tumor (66%), distant metastases (20%) or lymph nodes (14%). 89% were histological samples and 11% cytological samples.

454 (90%) patients discontinued treatment due to disease progression (51%), immuno-related adverse events (irAEs) (17%) or treatment completion after 35 cycles (13%). 445 patients (90%) had progressive disease at time of data cut-off, and 39 patients (8%) continued pembrolizumab beyond progression due to clinical benefit and 28 patients (6%) were rechallenged with immunotherapy in further lines. At time of data cut off, 331 patients (67%) had died, with 228 (69%) due to progression of disease.

**Figure 1:**
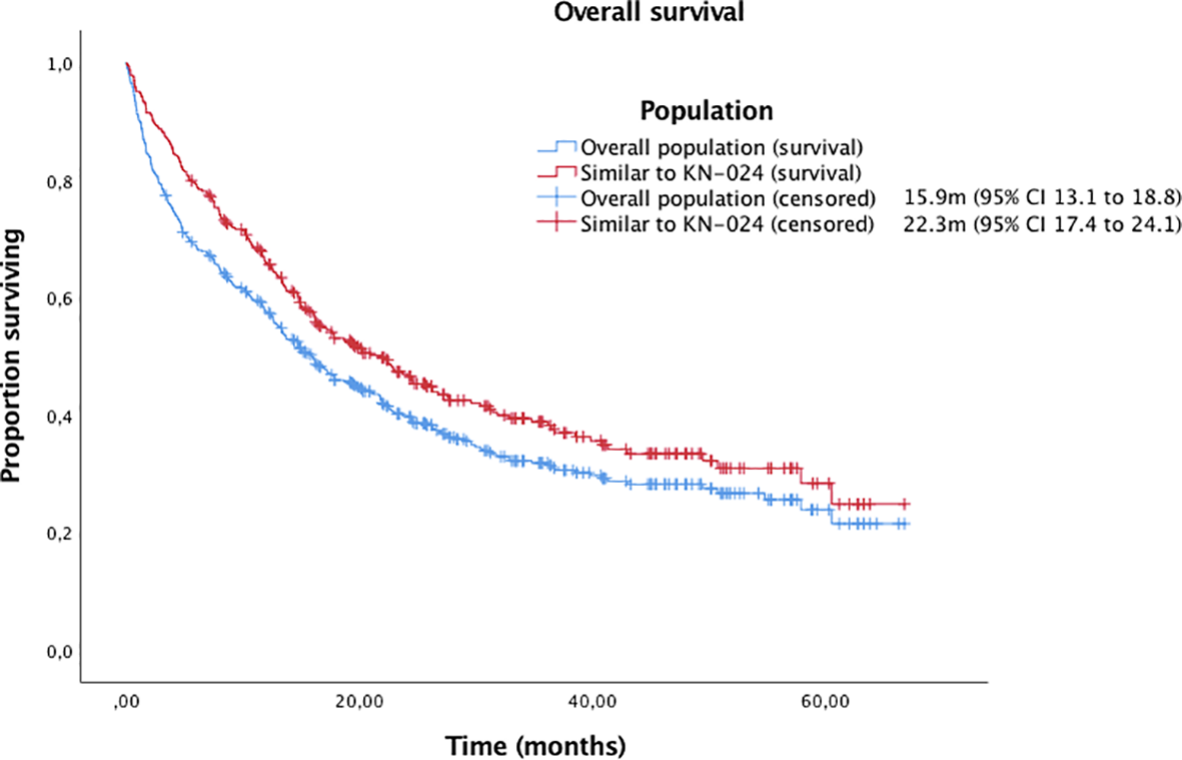
Kaplan-Meier curves for OS in the overall population and patients with similar inclusion criteria to Keynote-024 trial.

**Figure 2:**
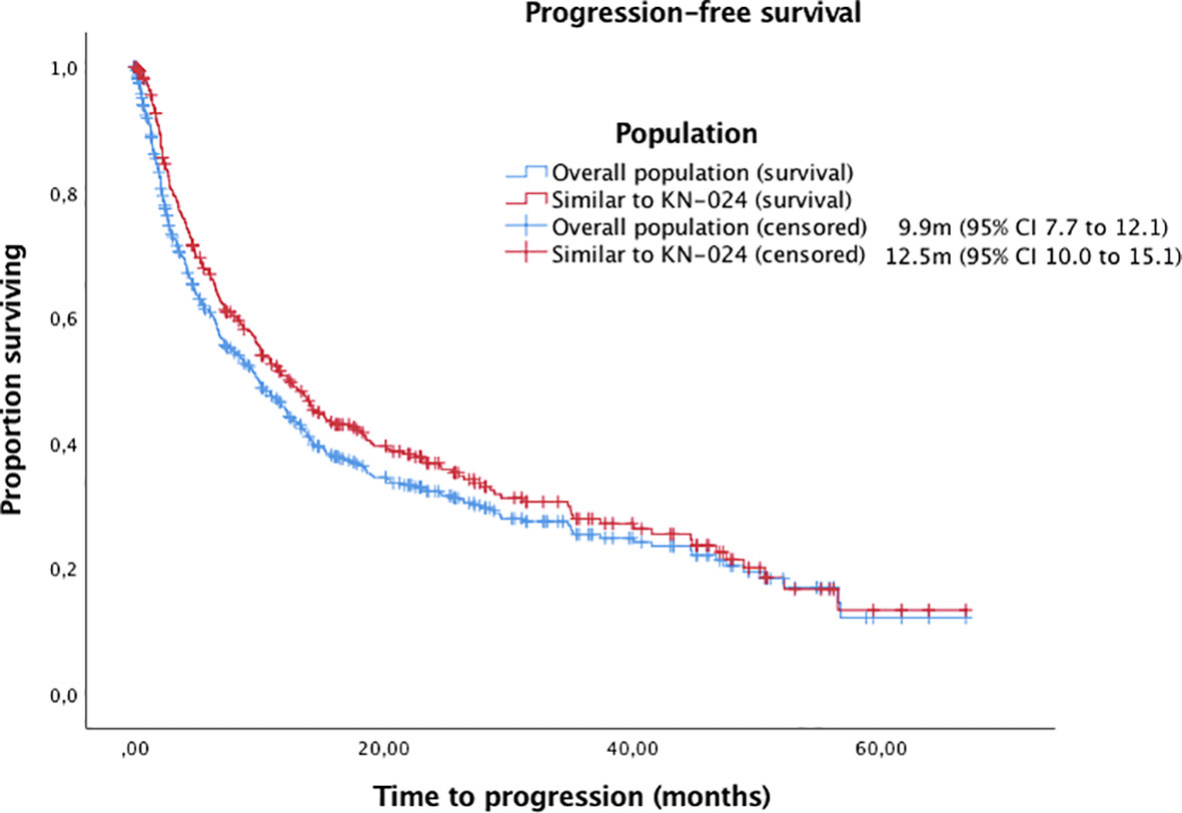
Kaplan-Meier curves for PFS in the overall population and patients with similar inclusion criteria to Keynote-024 trial.

With a median follow-up of 14.22 months (95% IC 12.5-16.0, IQR 23,13), the median OS and PFS were 15.9m (95% CI 13.1 to 18.8) and 9.9m (95% CI 7.7 to 12.1), respectively (**Figures 1**, **2**). Of 444 patients (90%) evaluable for response, 31 patients (6%) had complete response, 184 patients (37%) partial response, 112 patients (23%) stable disease and 117 patients (24%) progressive disease. ORR for the overall population was 43%.

**Figure 3:**
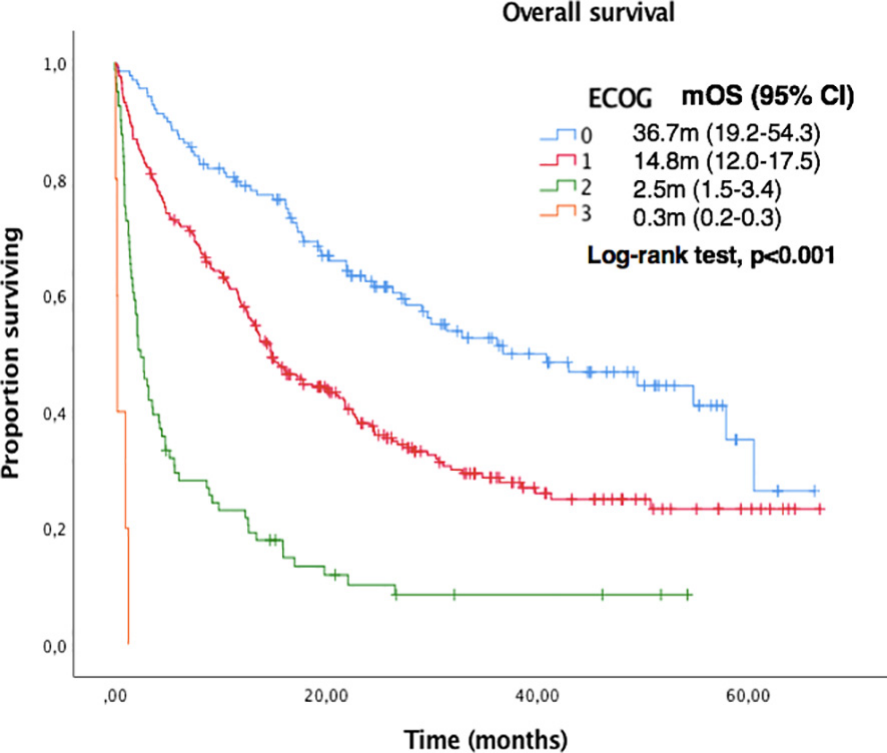
Kaplan-Meier curves for OS according to ECOG-PS in the overall population.

**Figure 4:**
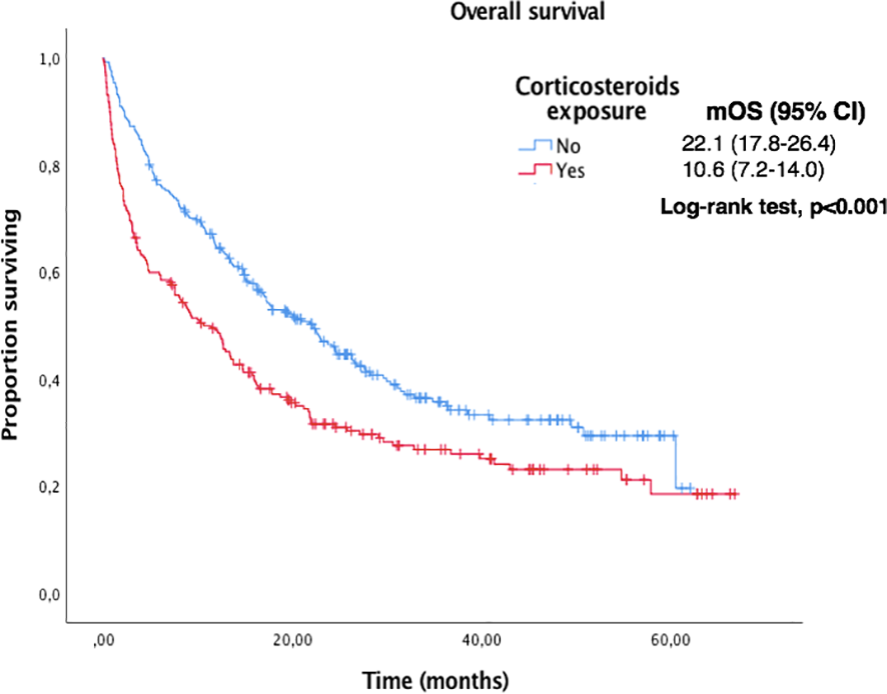
Kaplan-Meier curves for OS according to corticosteroids exposure in the overall population.

**Figure 5:**
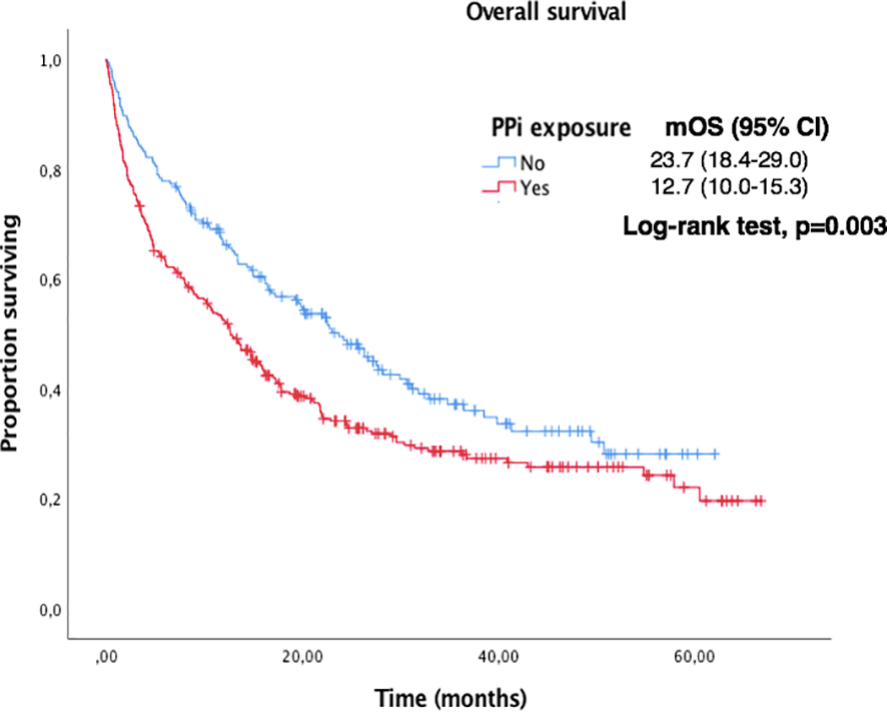
Kaplan-Meier curves for OS according to PPi exposure in the overall population.

**Figure 6:**
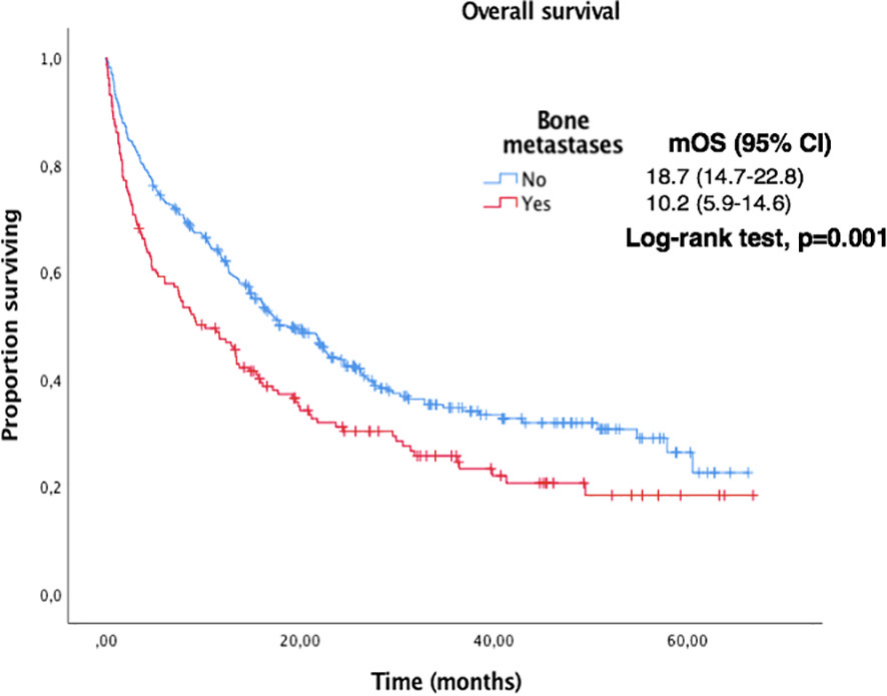
Kaplan-Meier curves for OS according to the presence of bone metastases in the overall population.

Median OS and PFS according to clinicopathological characteristics are shown in **Table 2**. ECOG-PS, corticosteroid exposure, PPi exposure and bone metastases were associated with shorter OS and were therefore included in a multivariate model. **Figures 3**–**6** show survival curves according to different prognostic factors. An interaction between ECOG-PS and corticosteroid exposure was also observed in the multivariate analysis for OS: corticosteroid exposure (HR 1.5, 95% CI 1.1 to 1.8) and ECOG-PS (HR 2.4, 95% CI 2.0 to 2.8) (**Table 3**). We did not find a statistically significant association between survival outcomes and age, sex, smoking status, AB exposure nor PD-L1 status.

**Table 2:** Survival outcomes according to clinicopathological characteristics.

	Median OS - months (95% CI)	p-value (univariate analysis)	Median PFS - months (95% CI)	p-value (univariate analysis)
Age				
<75	16.5 (12.4-20.6)	0.069	10 (7.5-12.4)	0.829
≥75	12.7 (7.8-17.6)		10.2 (6.8-13.5)	
Sex				
Male	15.5 (13-18)	0.968	10.0 (7.4-12.6)	0.351
Female	17.8 (10.6-25.1)		9.8 (6.9-12.8)	
Race				
White	15.9 (13.0-18.8)	0.679	9.9 (7.7-12.1)	0.850
Asian	39.8 (0-NR)		24.6 (0-60.2)	
Black	14.7 (0-NR)		3.1 (0-NR)	
Smoking habit				
Former smoker	22.3 (11.8-32.8)	0.362	6.6 (3.5-9.7)	0.155
Current smoker	15 (12-18)		11.7 (8.9-14.5)	
Never smoker	14.8 (9.1-20.4)		9.4 (6.3-12.4)	
BMI				
<18,5	5.2 (0.0-13.2)	0.089	8.5 (0.1-17.0)	0.026
18,5-24,9	15.5 (10.8-20.1)		7.2 (4.1-10.2)	
≥25	17.3 (13.2-21.4)		13.4 (10.6-16.2)	
ECOG-PS				
0	36.7 (19.2-54.3)	< 0.001	14.4 (9.6-19.1)	< 0.001
1	14.8 (12.0-17.5)		10.8 (8.4-13.3)	
2	2.5 (1.5-3.4)		2.5 (0.5-4.5)	
3	0.3 (0.2-0.3)		0.3 (0.0-1.3)	
AB exposure				
No	15.7 (12.2-19.3)	0.984	9.1 (6.7-11.4)	0.382
Yes	16.2 (10.9-21.6)		12.3 (9.4-15.1)	
Corticosteroid exposure				
No	22.1 (17.8-26.4)	< 0.001	10.1 (7.5-12.7)	0.082
Yes	10.6 (7.2-14.0)		9.3 (5.7-13.0)	
Reason for treatment with corticosteroids				
irAEs	NR (0.0-NR)	< 0.001	24.6 (8.8-40.5)	< 0.001
Management of comorbidities/symptom	4.7 (1.6-7.8)		4.5 (1.8-7.3)	
PPi exposure				
No	23.7 (18.4-29.0)	0.003	11.5 (7.6-15.4)	0.041
Yes	12.7 (10.0-15.3)		8.5 (5.8-11.3)	
Bone metastases				
No	18.7 (14.7-22.8)	0.001	11.1 (8.8-13.3)	0.164
Yes	10.2 (5.9-14.6)		7.3 (5.2-9.4)	
CNS metastases				
No	16.2 (13.0-19.4)	0.164	10.4 (8.0-12.8)	0.233
Yes	13.1 (5.7-20.6)		7.2 (3.5-11.0)	
Liver metastases				
No	16.3 (12.7-19.8)	0.393	11.0 (8.3-13.6)	0.418
Yes	12.9 (8.0-17.8)		7.2 (5.0-9.4)	
PD-L1 expression				
≥50-≤60	14.7 (9.6-19.8)	0.645	7.7 (5.3-10.1)	0.134
>60-<90	16.2 (9.9-22.4)		10.9 (8.1-13.7)	
≥90	15.9 (10.6-21.3)		11.7 (7.6-15.8)	
Best response				
CR	57.8 (51.9-63.8)	<0.001	46.7 (33.8-59.6)	<0.001
PR	NR (0.0-NR)		26.5 (20.0-33.0)	
SD	15.2 (13.2-17.1)		9.7 (7.6-11.8)	
PD	4.0 (3.1-5.0)		1.7 (1.4-2.1)	

OS, overall survival; PFS, progression-free survival; CI, confidence interval; BMI, body mass index; ECOG-PS, Eastern Cooperative Oncology Group-Performance Status; AB, antibiotic; irAEs, immune-related adverse events; PPI, proton pump inhibitor; CNS, central nervous system; PD-L1, programmed death-ligand 1; CR, complete response; PR, partial response; SD, stable disease; PD, progressive disease; NR, not reached.

**Table 3:** Analysis of prognostic factors for OS.

	Univariate HR (95% CI)	p-value	Multivariate HR (95%CI)	p-value
**ECOG-PS**	2.4 (2.0-2.9)	<0.001	2.4 (2.0-2.8)	<0.001
**Corticosteroids exposure**	1.5 (1.2-1.9)	<0.001	1.5 (1.1-1.8)	0.002
**PPi exposure**	1.4 (1.1-1.8)	0.03	1.2 (0.9-1.5)	0.200
**Bone metastases**	1.5 (1.2-1.8)	0.001	1.1 (0.9-1.4)	0.378

OS, overall survival; HR, hazard ratio; CI, confidence interval; ECOG-PS, Eastern Cooperative Oncology Group-Performance Status; PPi, proton pump inhibitors.

Type of antibiotic and its indication are shown in **Table 4**. 175 (35%) patients received treatment with antibiotic: 64% received oral administration (64%) and 36% intravenous administration. The median OS in patients receiving oral antibiotics vs. those receiving intravenous antibiotics was 21.9m (95% CI 11.7 to 32.2) vs. 10.4m (95% CI 3.1 to 17.7), p=0.005. There was no significant difference in terms of survival between an antibiotic exposure ≤7 days (57%) or >7 days (43%).

**Table 4:** Type of antibiotic and indication.

	N	%
Type of antibiotic		
Penicillins	89	52
Fluoroquinolones	50	26
Sulfonamides	9	6
Carbapenems	8	5
Macrolides	5	3
Oxazolidones	4	2
Tetracyclines	3	2
Glycopeptides	2	1
Fosfomycine	1	<1
Lincomycins	1	<1
Others/unknown	3	2
Indication		
Superior tract respiratory infection	117	67
Abdominal infection	14	8
Urinary tract infection	9	5
Dental infection	5	3
Lung abscess	4	2
Skin sepsis	4	2
Empiema	3	2
Bacteriemia	3	2
Surgery profilaxis	3	2
Pneumocystis Jirovecii profilaxis	3	2
Surgical wound infection	2	1
Urinary tract sepsis	1	<1
Unknown	7	4

217 (44%) patients received oral (78%) or intravenous (20%) corticosteroids and presented shorter OS: the median OS in patients receiving corticosteroids vs. those who did not was 10.6 m (95% CI 7.2 to 14.0) vs. 22.1m (95% CI 17.8 to 26.4), p<0.001. The median OS in patients with oral vs. intravenous corticosteroids was 12.9m (95% CI 8.1 to 17.7) vs 3.52m (95% CI 0.0 to 8.4), p=0.001.

Finally, we analysed those patients with similar inclusion criteria to Keynote-024 trial (1, 2) (excluding patients with ECOG-PS≥2 or untreated brain metastases). Of the 329 patients (67%) included, the median OS and PFS were 22.3m (95% CI 17.4 to 24.1) and 12.5m (95% CI 10.0 to 15.1), respectively, with an ORR of 48%.

## Discussion

Our real-world study confirms that corticosteroid treatment and poor ECOG-PS are negative predictive factors for first-line pembrolizumab monotherapy in patients with advanced high PD-L1 NSCLC.

The negative impact of corticosteroids was observed when they were administered as symptomatic treatment or for concomitant diseases, but not for the management of irAEs. Corticosteroid treatment should only be used under necessary conditions. Our findings are in line to previously reported series in advanced NSCLC patients (16, 24) and in other solid tumors (25). However, there are also conflicting results regarding the detrimental effect of corticosteroids for irAEs (26, 27), so the impact of corticosteroids for the management of irAEs in survival outcomes remains unclear.

Bone metastases and treatment with PPi were found to be negative predictive factors in the univariate analysis but were not confirmed in multivariate analysis. Other potential factors such as smoking habit, liver or CNS metastases, BMI or older age were not found to have negative impact, although there was a trend to a shorter median PFS and OS. Those findings are in line to similar previous studies with immunotherapy in advanced NSCLC (16–18).

We did not find a negative impact of antibiotic exposure, nor BMI in outcomes of patients receiving pembrolizumab. Interestingly, this finding was also observed in patients receiving first line chemotherapy and immunotherapy (28, 29). Of note, survival outcomes were significantly better in patients receiving oral than intravenous antibiotics in the univariate analysis, probably because patients receiving intravenous antibiotics presented a more serious infection.

To date, PD-L1 expression is the only validated biomarker in advanced NSCLC although it has several limitations (30). However, outcomes do not always correlate with PD-L1 expression level, as observed in our study, even in patients with high PD-L1 expression (8). There is an urgent need to define new biomarkers to better select the best treatment option. In the absence of more accurate biomarkers, the combination of chemotherapy and immunotherapy might be a good alternative in patients with advanced high PD-L1 NSCLC with negative predictive factors (10–14, 31).

One important finding of our study is that survival outcomes were similar to those observed in the Keynote-024 (1, 2) when patients with comparable characteristics were analysed, that were significantly better than in the overall population. This finding reaffirms the fact that patient with worse conditions receive treatment outside clinical trials. Additionally, our patient cohort has many similarities with the biggest real-world cohort reported in terms of age, histology, ECOG-PS, bone metastases and smoking history (16). We report a median age of 67.3 years vs. 70.1.; 76% of non-squamous histology vs. 77.9%, 83% of ECOG-PS 0-1 vs. 82.6%, 32% of patients with bone metastases vs. 33.6 and 92% of former/current smokers vs. 89.2%. A surprising fact, however, is that in our cohort we did not find a negative impact of antibiotics and PPI exposure, although a higher exposure was observed in our series. These differences may be explained by a smaller sample size, a shorter follow up in our cohort, or the possible different data frames for considering concomitant medication exposure.

Our study presents some limitations. Due to the retrospective design, some inherent selection bias may be implied, although this should be minimized by the consecutive patient selection criteria. In addition, data collection may have been heterogeneous between different institutions and data frames of some treatments may not be exact. However, the optimal time to collect concomitant antibiotic exposure is unclear, so we considered the same treatment period proposed in previous series (16).

Finally, as already indicated, the sample size and the follow-up may have meant that some numerical trends and significance of univariate analyses on predictive factors could not be confirmed after multivariate analysis, as well as the survival data may be immature. Despite these limitations, our real-world study supports the available evidence provided by randomized trials and other real-world cohorts of the efficacy of first line pembrolizumab in patients with advanced high PD-L1 NSCLC.

In conclusion, our study reaffirms the efficacy of first line pembrolizumab monotherapy in patients with advanced NSCLC and high PD-L1 expression with similar outcomes to those previously reported. Patients receiving corticosteroid treatment and with ECOG-PS 2 present worse outcomes after multivariate analysis. Alternative treatment options may be explored for patients receiving detrimental concomitant medication or unfit patients.

## Data Availability

The original contributions presented in the study are included in the article/supplementary material. Further inquiries can be directed to the corresponding author.
